# Beyond the samples: Nigerian research staff perspectives on a multicentre neonatal sepsis study’s impact

**DOI:** 10.1038/s41467-025-62102-4

**Published:** 2025-07-22

**Authors:** Chinenye Akpulu, Rashida Yakubu, Fatima Tukur, Ashiru H. Yusuf, Aisha Kassim, Aisha Mukaddas, Adeola Adeleye, Fatima Alkali, Augustine O. Maduekwe, Queen Nsude, Vivian Asunugwo, Mary-Joe Okenu, Samuel Yakubu, Lamidi Audu, Mairami Amsa, Fatima Isa Modibbo, Kathryn Thomson, Chioma R. Achi, Kenneth C. Iregbu, Timothy R. Walsh, Kirsty Sands

**Affiliations:** 1 Department of Biology, Ineos Oxford Institute, University of Oxford, Oxford, UK; 2 National Hospital Abuja, Abuja, Nigeria; 3 Murtala Muhammad Specialist Hospital, Kano, Nigeria; 4 Bayero University Kano, Kano, Nigeria; 5 Wuse District Hospital, Abuja, Nigeria; 6 Debbo Diagnostic, Lagos, Nigeria; 7 Department of Infectious Disease Epidemiology and Dynamics, London School of Hygiene and Tropical Medicine, London, UK; 8 Infection and Immunity, School of Medicine, Cardiff University, Cardiff, UK

**Keywords:** Infectious-disease diagnostics, Outcomes research

## Abstract

Based on experiences of Nigerian healthcare staff in a multi-centre international neonatal sepsis study, the authors describe insights on capacity strengthening, patients’ engagement, and discuss long-term challenges in sustaining outcomes after project funding ends.

## Teaser

Drawing on experiences of Nigerian healthcare staff in a multi-centre international study, we describe insights into capacity strengthening, patients’ engagement, and discuss long-term challenges in sustaining outcomes after project funding ends.

## Healthcare challenges in Nigeria and neonatal sepsis research

Nigeria is the most populated country in Africa, with over 230 million people^1^ divided into 36 states across six geopolitical zones. Nigeria’s healthcare system is chronically underfunded and faces persistent shortages in healthcare professionals. Limited financial protection mechanisms leave much of the population reliant on out-of-pocket payments for healthcare^2^. Northern Nigeria, accounting for ~54% of the population, has some of the country’s worst healthcare indicators, particularly maternal, newborn, and infant mortality^3^. Premature birth remains the leading cause of neonatal mortality, alongside birth complications and neonatal infections^4^ with 27 neonatal deaths per 1000 live births reported in 2022^4^. Documented neonatal sepsis remains alarmingly high with substantial regional variation^5–7^.

## Burden of Antibiotic Resistance in Neonates from Developing Societies (BARNARDS)

BARNARDS (2015–2018) established a network across South Asia and Africa to investigate the impact of antimicrobial resistance (AMR) on neonatal morbidity and mortality in seven low- and middle-income countries (LMICs). By standardising blood cultures and implementing diagnostics, the study enabled bacterial identification and AMR profiling^8–14^. In Nigeria, research was coordinated by a local principal investigator, to support participating hospitals through the construction of wards, laboratories, provision of consumables, and recruitment of clinical/research staff. Teams comprised consultants, researchers, microbiologists, nurses and laboratory personnel, operating within an ethos of local ownership and interdisciplinary collaboration.

The second phase, BARNARDS-II (2023–2025), expanded to seven Nigerian hospitals investigating AMR, antimicrobial use (AMS), and healthcare economics for neonatal sepsis management. Research capacity strengthening programmes, however, raise important ethical considerations including sustainability, equity, and the avoidance of extractive practices, ensuring local clinical benefit^15–17^.

## Basis for reflections: insights and approach

This article was endorsed by the Nigerian BARNARDS investigators; its narration reflects the views of the Nigerian staff and was jointly analysed by both the Nigerian and UK-based authors.

Fifteen Nigerian research assistants, laboratory scientists, consultant neonatologists, and consultant clinical microbiologists from three tertiary and secondary healthcare study sites: Wuse District Hospital (WDH), National Hospital Abuja (NHA), and Murtala Mohammed Specialist Hospital, Kano (MMSH) participated via an information sheet outlining the intended use of their reflections. Following consent, a questionnaire was issued to explore participants’ roles, experiences and impacts of BARNARDS. Participants were encouraged to elaborate freely, and responses were submitted anonymously. Responses were analysed using inductive reflexive thematic analysis following Braun and Clarke (2006, 2021)^18,19^. Twenty-eight categories were synthesised into overarching themes and quality-checked using Braun and Clarke’s recommended criteria (Source Data file).

## BARNARDS impact

**Fig. 1:**
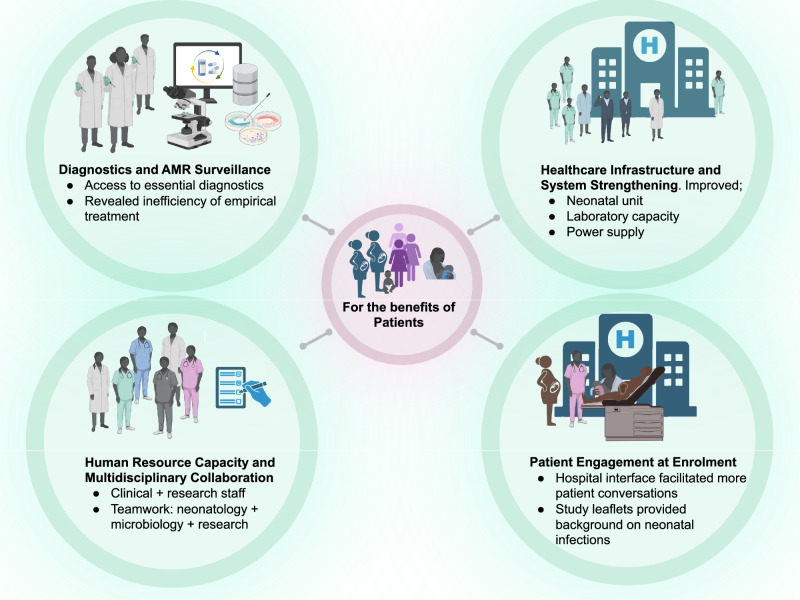
Perceived impact of BARNARDS. Research staff involved in the BARNARDS study reported perceived improvements in hospital infrastructure, enhanced clinical and laboratory capacity, better interdisciplinary collaboration, and increased opportunities for patient edification during enrolment. *Created in BioRender. Akpulu, C. (2025)* https://BioRender.com/hug34wh.

The staff reflected on the study’s influence beyond the research aims, particularly in clinical care and staff-patient interactions. The sustained presence and access to diagnostics encouraged greater engagement with mothers to support infant care and promote sepsis and infection prevention. Enhancements in clinical practice, institutional systems, and caregiver engagement underscored the multidimensional nature of the research impact in this setting, a point frequently mentioned by the participants (Fig. 1). Beyond immediate benefits to families, staff reported improvement in clinical skills, enhanced hospital workflows, and increased community awareness of neonatal health.

Attendance at a molecular microbiology workshop at the host institution facilitated longer-term capacity development, with staff returning to support local diagnostics and microbiology. Several staff transitioned from project implementation roles to research leadership, while others pursued further education, such as completing doctoral research.

## From diagnostics to community follow-up

During the project, free diagnostic tests enabled timely and accurate identification of pathogens and more effective implementation of AMS. As stated by an MMSH staff member, *“many lives were saved through this”*. Staff consistently reported a noticeable decline in neonatal deaths. At MMSH, the average mortality rate reduced from approximately 33% to 17% over the study period; however, we acknowledge that this change was not quantitatively measured. One consultant neonatologist noted, *“the gap between neonatology and clinical microbiology was narrowed,”* describing the ability to confirm sepsis on-site as a major advancement. Diagnostic access increased awareness of high rates of AMR to first-line treatments (ampicillin and gentamicin) recommended by the World Health Organisation (WHO)^12,20^, leading to more appropriate treatment decisions. One clinician remarked, “…*the discovery of the ineffectiveness of the combination of ampicillin and gentamicin…must have saved a lot of lives and must have been doing so even after the study”*. Outside of BARNARDS, blood culture typically cost $30–$35, prohibitively expensive for most families, especially in northern Nigeria, where salaries fall below the national average and poverty levels are high^21^. This financial barrier not only limited access to care but also contributed to an underestimation of the true burden of neonatal sepsis^12,20^.

Nigeria continues to face a critical shortage of healthcare workers, with a doctor-to-patient ratio of approximately 1:5000, far below the WHO’s recommendation of 1:600^22^. To partially address this, BARNARDS financed the employment of three full-time clinicians to supplement hospital staff. As families became more confident in receiving prompt and professional attention, staff reported a perceived increase in patient trust and return visits. A parallel integration of community-recruited research assistants provided further support across patient enrolment, laboratory workflows, and follow-up. At MMSH, >6000 mother-infant dyads were enroled, many of whom lacked reliable contact information. Research assistants had to travel long distances to locate families and ensure complete follow-up “*the execution of the project was seamless due to the passion and dedication of research assistants”*. Clinical staff reflected further, to say *“follow up of hundreds of women around Kano metropolis was only made possible due to the tenacity of the research assistants”*.

## Multidisciplinary team dynamics

**Fig. 2:**
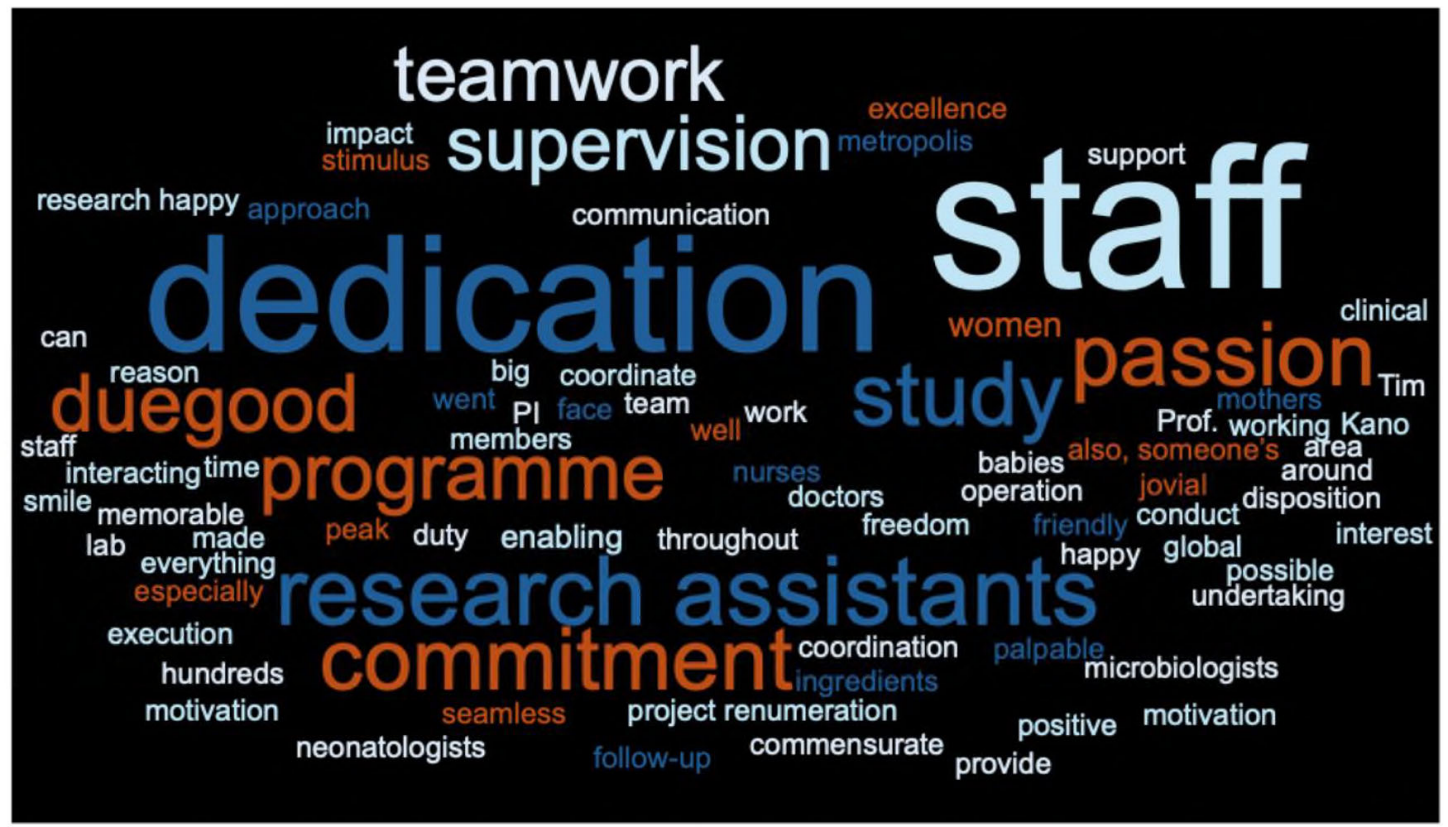
A word cloud generated from staff discourse. Word cloud generated from staff reflections related to research conduct (Source Data file). Terms reflect how participants described team culture and ethical approaches to clinical research delivery.

Staff highlighted importance of strong leadership, open communication, and coordination across clinical, laboratory, and research roles (Fig. 2). While adherence to protocol was essential, staff emphasised that successful research depended equally on team unity, motivation, and adaptability. Staff described challenges of high patient admissions, limited infrastructure, and overlapping responsibilities which were ultimately resolved by problem-solving, and a strong sense of collective purpose. Terms such as “passionate,” “dedicated,” “friendly,” and “jovial” were frequently used to capture the team’s ethos.

## Sustaining diagnostics

Sustaining diagnostic capacity in resource-limited settings requires dependable access to infrastructure, equipment, and consumables. Site-specific assessments identified clinical and infrastructural needs of each participating neonatal unit and laboratory permitting targeted support for the study objectives, and longer-term improvements in neonatal healthcare.

At one hospital, limited bed space had previously forced staff to turn away neonates. A participant explained: *“Renovation of a wing at the SCBU, construction of a laboratory, new baby cots, incubators, resuscitaires, and additional bed capacity provided for SCBU and maternity unit, all of which provided better facilities and care environment for the neonates”*.

BARNARDS-II has completed building a permanent laboratory replacing the previously stand-alone laboratory constructed from a shipping container. The new laboratory, solar-powered to enhance sustainability, is integrated into the hospital and will be formally handed over to the hospital after study completion. These locally-inspired interventions demonstrate the quintessential value of modest but targeted investments, strengthening local research and sustainable clinical services in resource-constrained settings.

Despite technological advancements, unreliable and irregular electricity supply remains a significant barrier across sub-Saharan Africa, negating the operation of diagnostic equipment^23–25^. BARNARDS supplemented the national grid in MMSH with solar power panels and capacitors; regrettably, it was insufficient to meet the continuous power demands of essential equipment (BACTEC™), resulting in diagnostic delays. As emphasised, by Okeke (2011) weaknesses in energy and laboratory infrastructure continue to undermine both clinical services and efforts to expand access to reliable diagnostics across the region^25^.

## Exit strategies: ethical implications of withdrawal

Although externally funded projects like BARNARDS provide temporary relief and infrastructural improvements, they also create new dependencies. Without careful planning, project exiting can result in abrupt withdrawal of critical resources, leading to disruptions in care, loss of trust, and exacerbation of healthcare inequities.

In BARNARDS-I, diagnostic capacity was introduced, yet sustainability remained a persistent challenge. After project closure, equipment including the BACTEC™ became inoperable due to funding gaps, negating access to life-saving diagnostics. Communities that previously benefited from enhanced care, experienced frustration and disillusionment when services ceased due to project closure. These pertinent experiences emphasise the importance of including exit strategies within externally funded research projects. Long-term partnerships with governments, transition strategies, and sustainability planning should be integral components to ensure continued patient care beyond the research project.

While this article reflects experiences of staff involved in BARNARDS, we acknowledge absence of perspectives from other stakeholders including patients, or community leaders; nonetheless, these reflections offer valuable insight into operational and ethical dimensions of research in this setting.

## Conclusion

Our experience in BARNARDS highlights the need for funders, policymakers, and health institutions to incorporate sustainability planning from project outset. Partnerships with national governments, targeted investment in clinical microbiology infrastructure, and capacity development planning are essential to ensure sustainability for diagnostics and treatment beyond the lifespan of project. The absence of structured exit strategies in externally funded research risks undermining community trust and weakening future research engagement. Although this perspective focuses on Nigerian sites, similar sustainability challenges likely exist across the BARNARDS network, warranting further investigation.

BARNARDS-II offers an important opportunity of reflection to address these pertinent issues, particularly sustainability, local ownership, and long-term capacity strengthening. Imperative ethical questions regarding who is responsible for sustainability in LMICs with constrained healthcare resources must be fully addressed.

## Source data

Source Data

## Data Availability

All data supporting the narrative this article are included within the manuscript. Source data are provided with this paper.
